# On the formalism of the screening paradox

**DOI:** 10.1371/journal.pone.0256645

**Published:** 2021-09-01

**Authors:** Jacques Balayla

**Affiliations:** Department of Obstetrics and Gynecology, McGill University, Montreal, Quebec, Canada; Roswell Park Cancer Institute, UNITED STATES

## Abstract

Bayes’ Theorem imposes inevitable limitations on the accuracy of screening tests by tying the test’s predictive value to the disease prevalence. The aforementioned limitation is independent of the adequacy and make-up of the test and thus implies inherent Bayesian limitations to the screening process itself. As per the WHO’s *Wilson* − *Jungner* criteria, one of the prerequisite steps before undertaking screening is to ensure that a treatment for the condition screened for exists. However, when applying screening programs in closed systems, a paradox, henceforth termed the “screening paradox”, ensues. If a disease process is screened for and subsequently treated, its prevalence would drop in the population, which as per Bayes’ theorem, would make the tests’ predictive value drop in return. Put another way, a very powerful screening test would, by performing and succeeding at the very task it was developed to do, paradoxically reduce its ability to correctly identify individuals with the disease it screens for in the future—over some time *t*. In this manuscript, we explore the mathematical model which formalizes said screening paradox and explore its implications for population level screening programs. In particular, we define the number of positive test iterations (PTI) needed to reverse the effects of the paradox. Given their theoretical nature, clinical application of the concepts herein reported need validation prior to implementation. Meanwhile, an understanding of how the dynamics of prevalence can affect the PPV over time can help inform clinicians as to the reliability of a screening test’s results.

## 1 Introduction

### 1.1 Bayes’ Theorem and predictive values

Bayes’ Theorem describes the probability of an event occurring based on prior knowledge of conditions related to that specific event [1]. The essence of the Bayesian approach is to provide a mathematical model explaining how existing beliefs change in light of new evidence [2]. Remarkably, Bayes theorem’ has applications in innumerable fields. Not surprisingly, it has significant implications in epidemiological modelling as well. From Bayes’ theorem, we can derive the positive predictive value *ρ*(*ϕ*) (PPV) of a screening test, defined as the percentage of patients with a positive screening test that do in fact have the disease screened for, as follows [1]:

$$\begin{array}{c} \rho \left(\phi \right)=\frac{a\phi }{a\phi +(1-b)(1-\phi )} \end{array}$$ (1)

where *ρ*(*ϕ*) = PPV, a = sensitivity, b = specificity and *ϕ* = prevalence.

The PPV *ρ*(*ϕ*) is therefore a function of the disease prevalence, *ϕ*. As the prevalence increases, *ρ*(*ϕ*) also increases and vice-versa [3].

## 2 Bayesian dynamics of predictive values

Let us define a hypothetical disease present in a population. Said condition has a preclinical phase and is amenable to screening through a given test designed to detect it. The test therefore has all of the pertinent screening parameters—sensitivity, specificity, and negative and positive predictive values. Finally, as required by the WHO’s *Wilson* − *Jungner* criteria [4], a treatment for the condition screened exists. Let us denote *ϕ*_0_ as the original or initial prevalence before screening is undertaken. As per Eq (1), we thus obtain a positive predictive value *ρ*(*ϕ*_0_) at *t*_0_:

$$\begin{array}{c} \rho \left(\phi_{0}\right)=\frac{a\phi_{0}}{a\phi_{0}+\left(1-b\right)\left(1-\phi_{0}\right)} \end{array}$$ (2)

Assuming no new cases, it follows that as the individual with a positive screening test is treated, the disease prevalence *ϕ*_0_ drops by some magnitude *k*, which represents the percentage reduction in prevalence. Consequently, the predictive value *ρ*(*ϕ*) will drop by some factor as well, so that individuals who test positive at some time *t*_*k* > 0_ experience a positive predictive value of:

$$\begin{array}{c} \rho \left(\phi_{0}-k\right)=\frac{a(\phi_{0}-k)}{a\left(\phi_{0}-k\right)+\left(1-b\right)\left[1-\left(\phi_{0}-k\right)\right]} \end{array}$$ (3)

From the above equations, we define the ratio *ζ* as follows:

$$\begin{array}{c} \rho \left(\phi_{0}\right)>\rho \left(\phi_{0}-k\right)\Rightarrow \frac{\rho (\phi_{0}-k)}{\rho (\phi_{0})}=\zeta \left(\phi_{0},k\right) \end{array}$$ (4)

where 0 < *ζ* < 1 and *k* > 0. Given the shape of the screening curve, and the principle of the prevalence threshold, even small changes in the prevalence *ϕ* can have significant changes in the positive predictive value *ρ*(*ϕ*). To determine the degree of reduction in the predictive value of the screening test at time *t*_*k*_, we take the ratio of *ρ*(*ϕ*) at two different times, be it *t*_0_, and some later time *t*_*k*_ with a prevalence reduction of *ϕ*_0_ − *k*, where *k* < *ϕ* is the percentage reduction in prevalence:

$$\begin{array}{c} \frac{\rho (\phi_{0}-k)}{\rho (\phi_{0})}=\frac{\left(\phi -k)[a\phi +(1-b)(1-\phi )\right]}{\phi [a(\phi -k)+(1-b)(1+k-\phi )]} \end{array}$$ (5)

Since *ϕ*_0_ − *k* yields a new, lower prevalence *ϕ*_*k*_, we can re-write the above equation as:

$$\begin{array}{c} \frac{\rho (\phi_{k})}{\rho (\phi_{0})}=\frac{\phi_{k}[a\phi_{0}+(1-b)(1-\phi_{0})]}{\phi_{0}[a\phi_{k}+(1-b)\left(1-\phi_{k}\right)]} \end{array}$$ (6)

Expanding the parentheses and simplifying the expression where the sum of the sensitivity *a* and specificity *b* is defined by *ε* = *a* + *b*, we obtain:

$$\begin{array}{c} \zeta \left(\phi_{0},k\right)=\frac{\rho (\phi_{k})}{\rho (\phi_{0})}=\frac{\phi_{k}\left(1-b\right)+\phi_{0}\phi_{k}\left(\varepsilon -1\right)}{\phi_{0}\left(1-b\right)+\phi_{0}\phi_{k}\left(\varepsilon -1\right)} \end{array}$$ (7)

The term *ε* − 1 = *a* + *b* − 1 has been previously defined in the context of receiver-operating characteristics (ROC) curves, and is termed the Youden’s *J* statistic [5]. As such, we can re-write the above equation as:

$$\begin{array}{c} \zeta \left(\phi_{0},k\right)=\frac{\rho (\phi_{k})}{\rho (\phi_{0})}=\frac{\phi_{k}\left(1-b\right)+J\phi_{0}\phi_{k}}{\phi_{0}\left(1-b\right)+J\phi_{0}\phi_{k}} \end{array}$$ (8)

From the above relationship, we infer:

$$\begin{array}{c} \underset{k\to 0}{\text{lim}}\frac{\rho (\phi_{k})}{\rho (\phi_{0})}=\underset{k\to 0}{\text{lim}}\frac{\rho (\phi_{0}-k)}{\rho (\phi_{0})}=\underset{k\to 0}{\text{lim}}\zeta =1 \end{array}$$ (9)

*ζ*(*ϕ*_0_, *k*) may be considered as the predictive value percentage loss as the prevalence decreases from *ϕ*_0_ to *ϕ*_*k*_. If we consider both *ϕ*_0_ and *k* as independent variables, both of which affect *ρ*(*ϕ*), we can establish the individual contributions of each variable towards *ρ*(*ϕ*) relative to each other through the partial differential equation *ζ*(*ϕ*_0_, *k*). To apply this equation in cases where the prevalence increases over time, simply flip the terms in the fraction to obtain the complement of *ζ*(*ϕ*_0_, *k*).

## 3 The prevalence threshold (*ϕ*_*e*_)

We previously defined the prevalence threshold as the prevalence level in the screening curve below which screening tests drops most precipitously [1]. In technical terms, this is equivalent to the inflection point in the screening curve below which the the rate of change of a test’s positive predictive value drops at a differential pace relative to the prevalence [1]. This value, termed *ϕ*_*e*_, is defined at the following point on the pre-test probability, or prevalence, axis:

$$\begin{array}{c} \phi_{e}=\frac{\sqrt{a(-b+1)}+b-1}{\left(a+b-1\right)}=\frac{\sqrt{a(-b+1)}+b-1}{\left(\varepsilon -1\right)} \end{array}$$ (10)

The corresponding positive predictive value is given by plotting the above equation into the positive predictive value equation (Eq 3):

$$\begin{array}{c} \rho \left(\phi_{e}\right)=\sqrt{\frac{a}{1-b}}[\frac{\sqrt{a(-b+1)}+b-1}{\left(\varepsilon -1\right)}] \end{array}$$ (11)

Interestingly, the above expression leads to the well known formulation for the positive predictive value as a function of prevalence and the positive likelihood ratio (LR+), defined as the sensitivity *a* over the compliment of the specificity *b* [6].

$$\begin{array}{c} \rho \left(\phi_{e}\right)=\phi_{e}\sqrt{\frac{a}{1-b}} \end{array}$$ (12)

### 3.1 Using radical conjugates to obtain the Youden’s J-independent equation for *ϕ*_*e*_

We can use radical conjugates to further simplify the prevalence threshold equation. Let c = 1-b, the complement of the specificity, otherwise known as the fall-out or false positive rate (FPR). The *ϕ*_*e*_ equation thus becomes:

$$\begin{array}{c} \phi_{e}=\frac{\sqrt{ac}-c}{a-c} \end{array}$$ (13)

Multiplying by its radical conjugate, we obtain:

$$\begin{array}{c} \phi_{e}=\frac{\sqrt{ac}-c}{a-c}[\frac{\sqrt{ac}+c}{\sqrt{ac}+c}] \end{array}$$ (14)

The square difference in the numerator yields:

$$\begin{array}{c} \phi_{e}=\frac{ac-c^{2}}{a\sqrt{ac}+ac-c\sqrt{ac}-c^{2}} \end{array}$$ (15)

Factoring out c, and knowing that $\frac{\sqrt{x}}{x}$ is equal to $\frac{1}{\sqrt{x}}$ we obtain:

$$\begin{array}{c} \phi_{e}=\frac{a-c}{\frac{a}{c}\sqrt{ac}+a-\sqrt{ac}-c}=\frac{a-c}{a\frac{\sqrt{a}}{\sqrt{c}}-\sqrt{ac}+a-c} \end{array}$$ (16)

Factoring out $\sqrt{ac}$ in the denominator’s first terms leads to:

$$\begin{array}{c} \phi_{e}=\frac{a-c}{\sqrt{ac}\left(\frac{a}{c}-1\right)+a-c} \end{array}$$ (17)

Finally, replacing 1 by c/c and factoring out the ensuing a-c term, we obtain:

$$\begin{array}{c} \phi_{e}=[\frac{a-c}{a-c}]\frac{1}{\frac{\sqrt{a}}{\sqrt{c}}+1} \end{array}$$ (18)

And thus, replacing c by 1-b the simplified version of the equation follows:

$$\begin{array}{c} \phi_{e}=\frac{\sqrt{1-b}}{\sqrt{a}+\sqrt{1-b}} \end{array}$$ (19)

**Fig 1:**
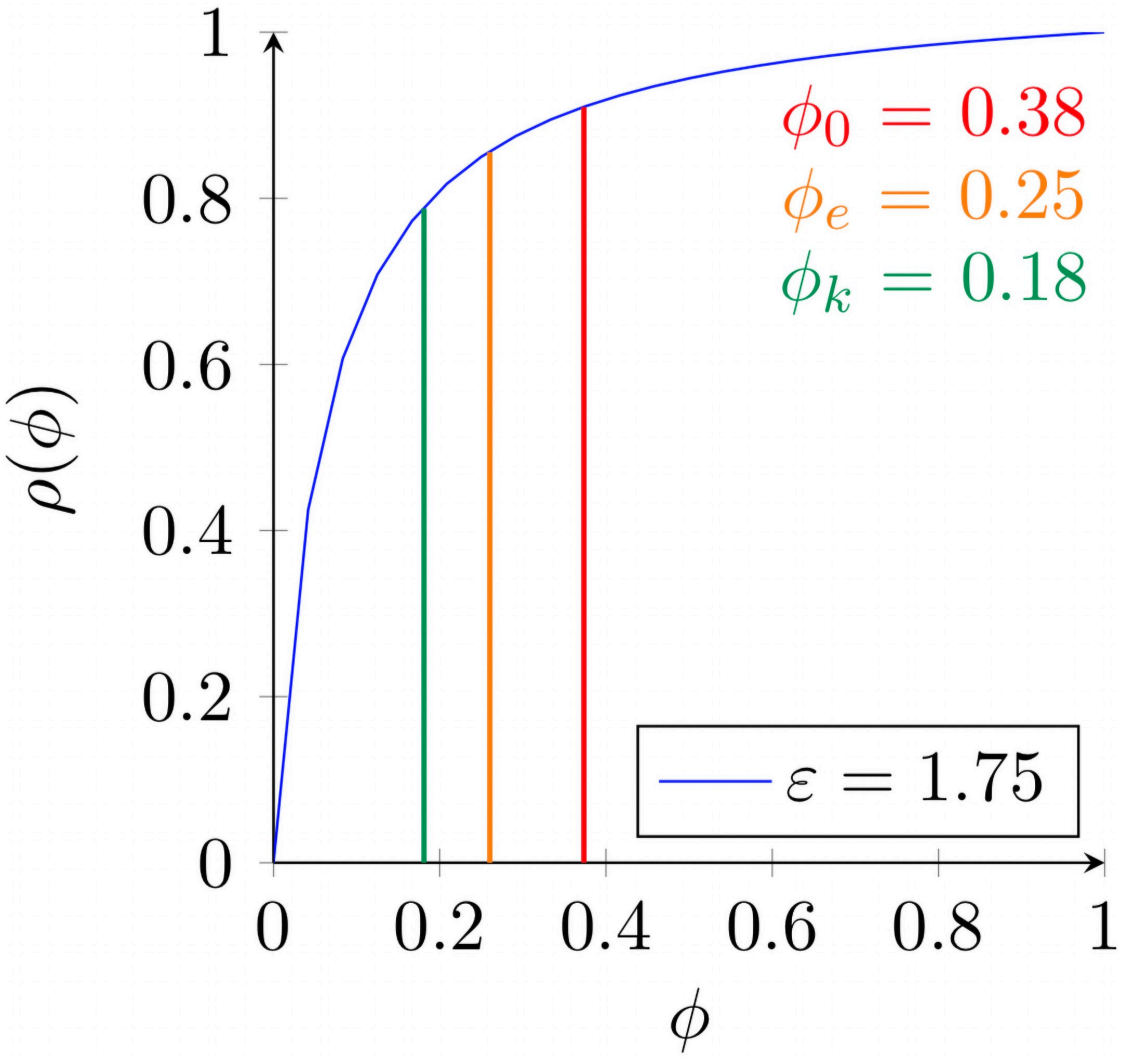
Example illustration of *ϕ*_0_, *ϕ*_*k*_, and *ϕ*_*e*_ where sensitivity a = 0.85, and specificity b = 0.90.

From the above relationship, we can take a hypothetical scenario and identify three different points in the screening curve, notably *ϕ*_0_—the initial prevalence, *ϕ*_*k*_—the prevalence at subsequent time k, and *ϕ*_*e*_—the prevalence threshold (Fig 1).

## 4 Relationship between *ϕ*_0_, *ϕ*_*k*_, and *ϕ*_*e*_

We can deduce important relationships between *ϕ*_0_, *ϕ*_*k*_, and *ϕ*_*e*_ that contextualize the screening paradox. First, we observe that though by definition *ϕ*_0_ > *ϕ*_*k*_, *ϕ*_*e*_ can either be outside or in between *ϕ*_0_ and *ϕ*_*k*_. As such three different scenarios may arise. Herein we explore each.

### 4.1 First scenario: *ϕ*_*e*_ > *ϕ*_0_ > *ϕ*_*k*_

By design, *ϕ*_0_ > *ϕ*_*k*_. Let *ϕ*_*e*_ define the prevalence threshold such that *ϕ*_*e*_ > *ϕ*_0_ > *ϕ*_*k*_. It then follows that *ϕ*_*e*_ − *ϕ*_*k*_ > *ϕ*_0_ − *ϕ*_*k*_ and thus *ϕ*_*e*_ − *ϕ*_0_ > 0. Since *ϕ*_0_ = *ϕ*_*k*_ + *k*, we obtain *ϕ*_*e*_ − *ϕ*_*k*_ − *k* > 0 or otherwise stated, *ϕ*_*e*_ > *ϕ*_*k*_ + *k* and thus *ϕ*_*e*_ − *ϕ*_*k*_ > *k*. We thus infer that:

$$\begin{array}{c} \underset{k\to \phi_{0}}{\text{lim}}\zeta \left(\phi \right)∼0 \end{array}$$ (20)

and

$$\begin{array}{c} \underset{k\to 0}{\text{lim}}\zeta \left(\phi \right)∼1 \end{array}$$ (21)

### 4.2 Second scenario: *ϕ*_0_ > *ϕ*_*k*_ > *ϕ*_*e*_

This scenario is akin to the first scenario in that the prevalence threshold lies outside the range between *ϕ*_0_ and *ϕ*_*k*_. However, an important difference arises. While lim_*k*→0_ *ζ*(*ϕ*) ∼ 1, by design *ϕ*_*k*_ > *ϕ*_*e*_, and thus the maximum value that *k* can take cannot be greater than *ϕ*_0_ − *ϕ*_*e*_, as *ϕ*_*k*_ → *ϕ*_*e*_. We thus infer that:

$$\begin{array}{c} \underset{k\to \phi_{0}-\phi_{e}}{\text{lim}}\zeta \left(\phi \right)∼1 \end{array}$$ (22)

and

$$\begin{array}{c} \underset{k\to 0}{\text{lim}}\zeta \left(\phi \right)∼1 \end{array}$$ (23)

The above relationships follow since for *ϕ* > *ϕ*_*e*_ → *dζ*/*dϕ* ∼ 0, as per Eq (11).

### 4.3 Third scenario: *ϕ*_0_ > *ϕ*_*e*_ > *ϕ*_*k*_

Perhaps the most interesting scenario is one where *ϕ*_0_ > *ϕ*_*e*_ > *ϕ*_*k*_. By design *ϕ*_0_ > *ϕ*_*k*_. Let *ϕ*_*e*_ define the prevalence threshold such that *ϕ*_0_ > *ϕ*_*e*_ > *ϕ*_*k*_. It then follows that *ϕ*_0_ − *ϕ*_*k*_ > *ϕ*_0_ − *ϕ*_*e*_ and thus 0 > *ϕ*_*k*_ − *ϕ*_*e*_. Since *ϕ*_*k*_ = *ϕ*_0_ − *k*, we obtain 0 > *ϕ*_0_ − *k* − *ϕ*_*e*_ and thus:

$$\begin{array}{c} k>\phi_{0}-\phi_{e} \end{array}$$ (24)

We thus infer that:

$$\begin{array}{c} \underset{k\to \phi_{0}-\phi_{e}}{\text{lim}}\zeta \left(\phi \right)∼1 \end{array}$$ (25)

In other words, when the initial prevalence lies beyond the prevalence threshold, changes in prevalence such that *k* approaches the difference between *ϕ*_0_ − *ϕ*_*e*_, the ratio of positive predictive values as determined by the *ζ*(*ϕ*) function approaches 1. However, we can theorize a case where *k* is sufficiently large so that *ϕ*_*k*_ goes well below *ϕ*_*e*_ and therefore:

$$\begin{array}{c} \underset{k\to \phi_{0}}{\text{lim}}\zeta \left(\phi \right)∼0 \end{array}$$ (26)

## 5 The screening paradox at the population-level

**Fig 2:**
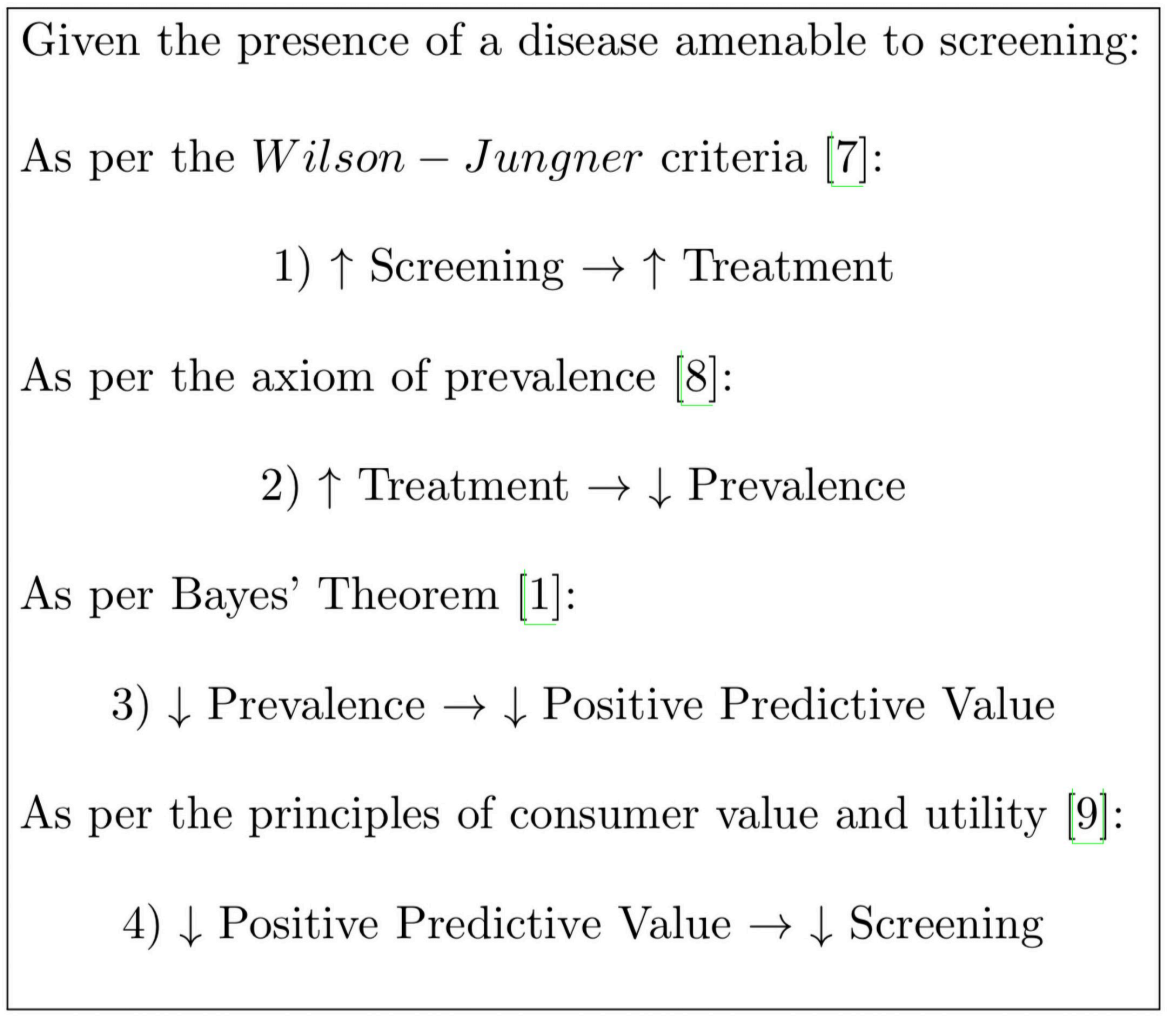
The process by which the screening paradox arises.

The mechanism by which the screening paradox arises is depicted through the following arrow flow diagram (Fig 2) [7–9].

Given the screening paradox, an increase in screening eventually leads to less, or more accurately, lower quality screening as the prevalence drops below the treshold. This paradox is inherently insurmountable unless acted upon by a subsequent test—either the same test repeated serially or an altogether different, better, diagnostic test [10] The calculation of such cumulative PPV is a form of Bayesian updating and can be explored in previous work by the author [10]. The salient points of that theory are further discussed next.

## 6 Overcoming the screening paradox

As the prevalence in a population drops with successful population-level screening and treatment, the positive predictive value of the screening test drops, and the false discovery rate, which is equivalent to the complement of the positive predictive value, increases. The aforementioned paradox occurs any time that disease is successfully treated because while *dρ*/*dϕ* drops throughout the function’s domain, it never reaches 0. In other words, the positive predictive value function always increases throughout its domain, so even minute changes in prevalence will bring about changes in the positive predictive value. That said, as we described above, the critical factor is where lie the initial prevalence level *ϕ*_0_, the subsequent prevalence level *ϕ*_*k*_, their difference *k*, and how they relate to the prevalence threshold, *ϕ*_*e*_, below which the screening paradox becomes more pronounced. In the presence of a screening paradox it is worth considering potential solutions to overcoming the losses in predictive value as the prevalence drops. Though many options exist, the most logical step would be to undertake serial testing, be it with the same test undertaken serially or an alternative test altogether. Herein we explore both scenarios.

### 6.1 Repeated testing with a single test

We have shown in previous work that a screening test carried out serially improves the overall positive predictive value when each individual test iteration is positive [10]. The number of serial iterations *n*_*i*_ required to achieve a desired *ρ*(*ϕ*) is given by the following ceiling function:

$$\begin{array}{c} n_{i}=\underset{\rho \to k}{\text{lim}}\lceil \frac{ln[\frac{\rho (\phi -1)}{\phi (\rho -1)}]}{ln[\frac{a}{1-b}]}\rceil \end{array}$$ (27)

The key question then becomes, how many serial positive tests are needed to mitigate or reverse the effect of the screening paradox when *ϕ*_*k*_ < *ϕ*_*e*_? In other words, to achieve a positive predictive value comparable to that under *ϕ*_0_? Given the geometry of the screening curve, the answer should be that the PPV ought to at least attain the level at the prevalence threshold as described in the third scenario in section 4 of this manuscript so that *ζ*(*ϕ*) ∼ 1. We can calculate the number of iterations $n_{i\phi_{e}}$ needed using the formula above, by plugging *ρ*(*ϕ*_*e*_) into *ρ* as defined in Eq (11), where $\rho \left(\phi_{e}\right)=\phi_{e}\sqrt{\frac{a}{1-b}}$.

$$\begin{array}{c} n_{i\phi_{e}}=\lceil \frac{ln[\frac{\phi_{e}\sqrt{\frac{a}{1-b}}\left(\phi -1\right)}{\phi (\phi_{e}\sqrt{\frac{a}{1-b}}-1)}]}{ln[\frac{a}{1-b}]}\rceil \end{array}$$ (28)

The above expression can be simplified by considering the square root of the positive likelihood ratio $\sqrt{\frac{a}{1-b}}$ as *ω*, and *ϕ*_*k*_ is the prevalence at subsequent time *k* such that:

$$\begin{array}{c} n_{i\phi_{e}}=\lceil \frac{ln[\frac{\omega \phi_{e}\left(\phi -1\right)}{\phi (\omega \phi_{e}-1)}]}{2ln\omega }\rceil =\lceil \frac{ln[\frac{\omega \phi_{e}\phi_{k}-\omega \phi_{e}}{\omega \phi_{e}\phi_{k}-\phi_{k}}]}{2ln\omega }\rceil \end{array}$$ (29)

We take the ceiling function of the above equation to ensure that we obtain an integer number of of positive test iterations (PTI) needed to surpass the prevalence threshold [10].

### 6.2 Using a different screening test

We can likewise revert the effects of the screening paradox by using a different screening test all together, which is the most common scenario in clinical practice today. That said, it would be impractical to determine the number of iterations of a different test for numerous reasons. First, different tests would have different sensitivity/specificity parameters, so a third test may be then needed in the rare scenario where two different positive ones are insufficient—rendering the notion of iteration inadequate. Likewise, and perhaps more importantly, there may not be an alternative screening test for a particular condition altogether, so the above exercise may be moot.

## 7 An SIR model without vital dynamics

To better illustrate the ideas above, we can take an infectious disease we shall call X for simplicity’s sake. The condition need not be infectious in nature, but an infectious agent lends itself well to the application of the concepts herein described. To estimate how the prevalence of disease X changes over time in a community outbreak closed system, we can come up with a theoretical SIR model [11]. An SIR model is an epidemiological model that computes the theoretical number of people infected with a contagious illness in a closed population over time. The name of this class of models derives from the fact that they involve coupled ordinary differential equations relating the number of susceptible people S(t), number of people infected I(t), and number of people who have recovered R(t) over time *t* [12]. One of the simplest SIR models is the Kermack-McKendrick model [13]. The dynamics of an infectious epidemic, such as the case of X, are often much faster than the dynamics of birth and death due to other causes than X, therefore, birth and death are often omitted in simple compartmental models. Otherwise stated—the population remains relatively stable over time *t* so that the individual parameters can change, but the total number of individual remains stable, as follows:

$$\begin{array}{c} S(t)+I(t)+R(t)=N \end{array}$$ (30)

where N is some constant.

The SIR system can be expressed by the following set of ordinary differential equations:

$$\begin{array}{c} \frac{dS}{dt}=-\frac{\beta IS}{N} \end{array}$$ (31)

$$\begin{array}{c} \frac{dI}{dt}=\frac{\beta IS}{N}-\gamma I \end{array}$$ (32)

$$\begin{array}{c} \frac{dI}{dt}=\gamma I \end{array}$$ (33)

where *β* is the average infection rate, *γ* is the recovery rate, *S* is the proportion of susceptible population, *I* is the proportion of infected, *R* is the proportion of removed population (either by death or recovery), and *N* is the sum of the latter three. From the above equations we obtain the basic reproduction number (*R*_0_) as a ratio of infection-to-recovery rates [14]:

$$\begin{array}{c} R_{0}=\frac{\beta }{\gamma } \end{array}$$ (34)

The ensuing equation to determine the number of susceptible individuals as a function of time becomes:

$$\begin{array}{c} S\left(t\right)=S\left(0\right)e^{-R_{0}\left(R\left(t\right)-R\left(0\right)\right)/N} \end{array}$$ (35)

where S(0)and R(0) are the initial numbers of, susceptible and recovered/dead subjects, respectively.

**Fig 3:**
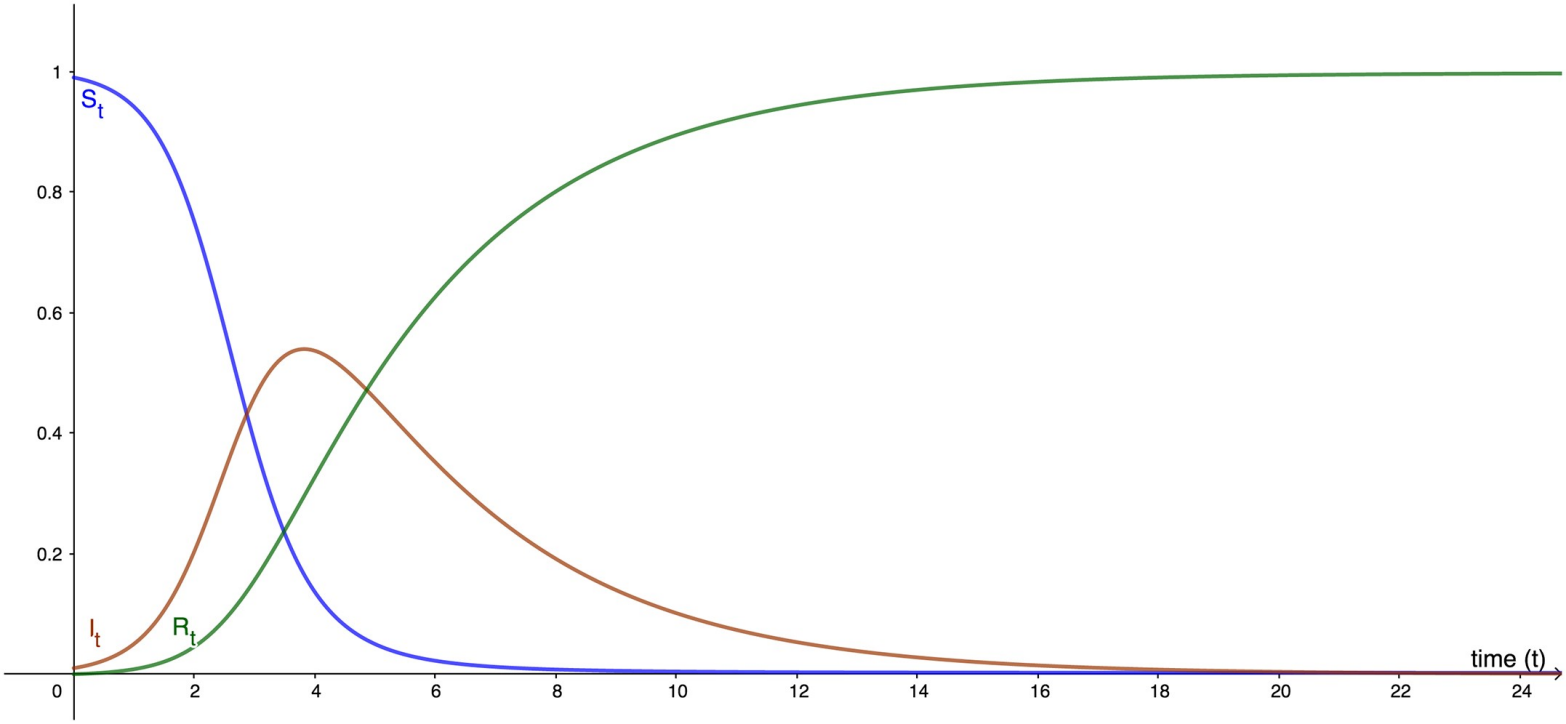
Dynamics of the SIR model.

The graphical representation of the above dynamics over some time *t* is seen in Fig 3.

**Fig 4:**
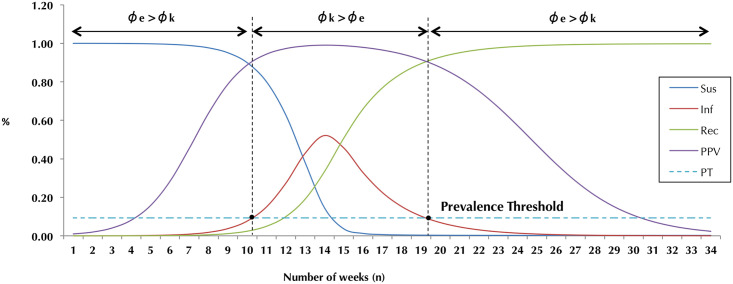
SIR + PPV: The SIR-P model.

For the purpose of the screening paradox, we need only focus on Eq 34, the rate of change of active infections *dI*/*dt*, which as a rate reflects the incidence of disease, but as an absolute value in a specific time *t* yields the prevalence of disease X at that point in time (Fig 4). We can use this value to determine how the PPV fluctuates over time. The differential equation relating the changes in PPV over time *t* therefore becomes:

$$\begin{array}{c} \rho \left(\phi ,t\right)=\frac{a[\frac{\beta IS}{N}-\gamma I]}{a\left[\frac{\beta IS}{N}-\gamma I\right]+\left(1-b\right)\left(1-\left[\frac{\beta IS}{N}-\gamma I\right)\right]}=\frac{a\frac{dI}{dt}}{a\frac{dI}{dt}+\left(1-b\right)\left(1-\frac{dI}{dt}\right)} \end{array}$$ (36)

Towards this end, let us assume that the condition X has a screening test with excellent sensitivity and specificity parameters of 95 and 99 percent, respectively. According to Eq 10, this test would have a prevalence threshold of 9.3 percent. Using this threshold, we can illustrate the full SIR model as follows (Fig 4).

Note the rise of the PPV (purple) as a function of prevalence (red). The delineation of the prevalence threshold (PT) at week 10 shows a corresponding flattening of the PPV, which holds steady almost horizontally. We thus observe that as the prevalence crosses the PT, the test performs well, with greater than 90 percent predictive value. However, once the prevalence drops below the PT once again, around week 19, the PPV begins to drop anew. Of note, this is a consequence of the success of the screening test in the first place—leading to the accurate detection of disease in a higher proportion of individuals once the prevalence threshold has been crossed and people being adequately treated or quarantined to prevent further spread. In other words—as stated before—by performing and succeeding at the very task it was developed to do, a screening test paradoxically reduces its predictive ability to correctly identify individuals with the disease it screens for in the future. The degree to which this paradoxical effect is observed depends on where we set our original prevalence, *ϕ*_0_, since *ζ*(*ϕ*) depends on both *ϕ*_0_ and *k*.

Likewise, we can use the SIR model to come demonstrate the dynamics of the epidemic of disease X in real-time, numerically, as observed in Table 1. The disease manifests over a number of weeks, affecting a peak 52 percent of this population by week 14. Because implicit to the paradox is the fact that *ϕ*_0_ > *ϕ*_*k*_, let us for argument’s sake take the maximum prevalence as the starting prevalence point *ϕ*_0_—though this need not be necessarily the case since the principles described in this work apply regardless of *ϕ*_0_. The time *k* thus corresponds to *ϕ*_*k*_ each subsequent week. The corresponding PPV and the ensuing *ζ*(*ϕ*_0_, *k*) values can be seen in Table 1. Note that since our test has a sensitivity of 0.95 and a specificity of 0.99, Youden’s *J* statistic equals 0.95+0.99−1 = 0.94. Finally, we take the ceiling function n iteration number to ensure that we obtain an integer number of positive test iterations (PTI) needed to surpass the prevalence threshold—thus enhancing the reliability of the screening process. Note that at the extremes of prevalence we would need to obtain 3 serial positive tests to achieve a PPV similar to that beyond the prevalence threshold. Once that threshold has been crossed, by definition $n_{i\phi_{e}}=1$. As noted above, other than developing newer, better screening tests, serial testing is one way to overcome the screening paradox [15]—be it with the same test done repeatedly or using a second, different diagnostic test altogether.

**Table 1:** Numerical representation of the SIR model.

Week (*t*)	PT	Susceptible	Infected	Recovered	PPV	*ζ*(*ϕ*_0_, *k*)	$n_{i\phi_{e}}$
1	0.093	0.9999	0.0001	0.0000	0.0094	0.0095	3.00
2	0.093	0.9998	0.0002	0.0000	0.0196	0.0198	3.00
3	0.093	0.9995	0.0004	0.0001	0.0405	0.0409	3.00
4	0.093	0.9988	0.0009	0.0003	0.0816	0.0824	3.00
5	0.093	0.9975	0.0020	0.0006	0.1577	0.1592	2.00
6	0.093	0.9946	0.0041	0.0012	0.2829	0.2857	2.00
7	0.093	0.9887	0.0087	0.0026	0.4541	0.4585	2.00
8	0.093	0.9764	0.0181	0.0055	0.6372	0.6433	2.00
9	0.093	0.9508	0.0376	0.0115	0.7878	0.7955	2.00
10	0.093	0.8993	0.0766	0.0241	0.8874	0.8960	2.00
11	0.093	0.8002	0.1502	0.0496	0.9438	0.9529	1.00
12	0.093	0.6271	0.2733	0.0997	0.9728	0.9822	1.00
13	0.093	0.3803	0.4289	0.1908	0.9862	0.9957	1.00
14	0.093	0.1454	0.5208	0.3337	0.9904	1.0000	1.00
15	0.093	0.0364	0.4563	0.5074	0.9876	0.9972	1.00
16	0.093	0.0125	0.3281	0.6594	0.9789	0.9884	1.00
17	0.093	0.0066	0.2246	0.7688	0.9649	0.9743	1.00
18	0.093	0.0044	0.1519	0.8437	0.9445	0.9536	1.00
19	0.093	0.0035	0.1022	0.8943	0.9154	0.9242	1.00
20	0.093	0.0030	0.0687	0.9284	0.8751	0.8835	2.00
21	0.093	0.0027	0.0461	0.9513	0.8210	0.8290	2.00
22	0.093	0.0025	0.0309	0.9666	0.7517	0.7590	2.00
23	0.093	0.0024	0.0207	0.9769	0.6676	0.6741	2.00
24	0.093	0.0023	0.0139	0.9838	0.5720	0.5775	2.00
25	0.093	0.0023	0.0093	0.9884	0.4713	0.4758	2.00
26	0.093	0.0022	0.0062	0.9915	0.3731	0.3768	2.00
27	0.093	0.0022	0.0042	0.9936	0.2847	0.2874	2.00
28	0.093	0.0022	0.0028	0.9950	0.2102	0.2123	2.00
29	0.093	0.0022	0.0019	0.9959	0.1512	0.1527	2.00
30	0.093	0.0022	0.0013	0.9966	0.1065	0.1076	2.00
31	0.093	0.0022	0.0008	0.9970	0.0739	0.0747	3.00
32	0.093	0.0022	0.0006	0.9973	0.0508	0.0512	3.00
33	0.093	0.0022	0.0004	0.9974	0.0346	0.0349	3.00
34	0.093	0.0022	0.0003	0.9976	0.0234	0.0236	3.00

Model for a screening test with 95% sensitivity and 99% specificity over time *t*.

PPV = positive predictive value, *ζ*(*ϕ*) is the PPV ratio between *ϕ*_0_ and *ϕ*_*k*_, N = number of iterations to overcome the screening paradox (S1 Data)

## 8 Conclusion

In this manuscript, we explore the mathematical model which formalizes the screening paradox and explore its implications for population level screening programs in the three possible scenarios—each as a function of the position of the initial prevalence of a condition relative to the prevalence threshold level of its screening test. Likewise, we provide a mathematical model to determine the predictive value percentage loss as the prevalence decreases and define the number of positive test iterations (PTI) needed to reverse the effects of the paradox when a single test is undertaken serially. Given their theoretical nature, clinical application of the concepts herein reported need validation prior to implementation. Meanwhile, an understanding of how the dynamics of prevalence can affect the PPV over time can help inform clinicians as to the reliability of a screening test’s results.

## Supporting information

S1 Data Supplemental file: Table data and graph. (XLSX)
